# A dataset exploring the substantial diversity of pesticide products: Insights from the UK and Ireland

**DOI:** 10.1016/j.dib.2024.110843

**Published:** 2024-08-14

**Authors:** Edward A. Straw, Dara A. Stanley

**Affiliations:** a School of Agriculture and Food Science, University College Dublin, Dublin, Ireland; b Department of Botany, Trinity College Dublin, Dublin, Ireland

**Keywords:** Pesticides, Pesticide diversity, Consumer pesticides, Professional pesticides

## Abstract

To understand the impacts of pesticides on non-targets, it is important to understand what pesticide products are authorised for use. Different pesticide formulations with the same active ingredient can pose different risks to non-target organisms due to the inclusion of co-formulants which can modify their toxicity. We collated datasets from the United Kingdom (UK) and Ireland containing information on all the authorised pesticide products (pesticide formulations and adjuvants). We reveal that there are 2,463 pesticide formulations authorised for use by professionals in the UK, representing 266 active ingredients. We also collated information on amateur pesticide formulations, finding 520 authorised in the UK, and adjuvants (separate products added to a tank mix to alter the spray action), finding 298 authorised in the UK. Although we focus on the UK dataset, the same overall trends are mirrored in the Irish data. For the first time we have brought together data on the diversity of pesticides on sale in the UK and Ireland.

Specifications Table

**Table:** 

Subject	Agronomy and Crop Science
Specific subject area	Ecotoxicology relating to the diversity of pesticide products used in farming as well as amenity and consumer uses.
Data format	Raw & Analyzed
Type of data	Table & Graph
Data collection	Pesticide product datasets were collected from national pesticide registration lists provided online by the UK and Irish governments. The data was cleaned and tabulated, with all extractable data summarised. Manual data cleaning was required to standardise the dataset; removing any spelling mistakes and correcting any inconsistent punctuation to ensure consistent reporting. Throughout only obvious spelling errors were corrected, and minor variations between data were assumed to be truthful variation.
Data source location	Data were collected from the UK and Ireland.
Data accessibility	Repository name: Zenodo Data identification number: https://zenodo.org/doi/10.5281/zenodo.13270718

## 1 Value of the Data

• This dataset reveals the considerable diversity of pesticides on sale and determines the most frequent active ingredients. This highlights to the ecotoxicological community what to study, allowing a data-led research approach.
• This is the first instance of a compiled database on pesticide product diversity. Previously the only data on this was held behind website interfaces, with no capacity for tabulation or synthesis of the data. Data from two separate regulatory regimes have been brought together for the first time.
• This dataset provides an accurate, citable, figure for the number of pesticide products available for purchase in the UK and Ireland. This is useful for ecotoxicological and agronomy research.
• The data is broken down by category: Professional and Amateur, as well as into pesticide classes and by producers. The number of products per active ingredient, or effective adjuvant component, has been calculated, revealing unforeseen trends in pesticide use.
• This dataset allows, for the first time, an international comparison of pesticide product diversity. The same processes were applied to both UK and Irish data, and data covers the same period, allowing a direct comparison between two nations of different sizes and different agricultural compositions.

## 2 Background

Pesticide users employ a range of pesticide products when controlling pests [1]. Not only a range of active ingredients used for different purposes, but also a range of formulations with the same active ingredients with subtly different uses. Pesticide formulations are mixtures of active ingredients (AI) (chemicals which confer pesticidal action) and co-formulants (chemicals which aid the active ingredient's function). Pesticide users can also modify their sprays with adjuvants (separate products added to a tank mix to alter the spray action) [2]. The chemicals in adjuvants which confer their intended function are called effective adjuvant components (EAC). Here we define the grouping of ‘pesticides products’ as both formulations and adjuvants. The diversity of active ingredients, formulations and adjuvants allows flexibility to use the right tool for the job.

A first step in beginning to understand the potential exposure of non-targets to pesticide formulations, co-formulants and adjuvants is to quantify the diversity of pesticide products on sale. Accordingly, we set out to collate information on the diversity of pesticide products and adjuvants in the UK and Ireland, so as to understand the potential diversity of co-formulants and adjuvants that non-targets could be exposed to.

## 3 Data Description

The article describes the dataset of UK and Irish pesticide products from 2021 to 2022 [3]. Pesticide product datasets were collected from national pesticide registration lists provided online by the UK and Irish governments. The data was cleaned and tabulated, with all extractable data summarised.

The dataset itself is available in .xlsx format, with multiple tabs for different data-subsets. The Summary Statistic Matrix details which metrics extracted were extracted from each dataset, detailing the comparability between nations. The Summary Statistics tab presents a synthesized and tabulated analysis of the raw datasets. Broken down by Nation, User Type and Category, the following information is extracted: number of formulations with multiple Active Ingredients; mean, median and range of products per Active Ingredient / Effective Adjuvant Component; the total number of products per Active Ingredient / Effective Adjuvant Component; the total number of products per function; the total number of products per Authorization holder.

Extracted and minimally cleaned data is then presented by Nation, User type and Category on individual tabs. See Table 1 for details.

**Table 1:** The different tabs for different datasets.

Nation	User Type	Category
Ireland	Amateur	Formulation
Ireland	Professional	Formulation
Ireland	Professional	Adjuvant
UK	Amateur	Formulation
UK	Professional	Formulation
UK	Professional	Adjuvant

## 4 Experimental Design, Materials and Methods

### 4.1 UK datasets

For pesticide formulations, the UK Chemical Regulation Division currently authorised pesticide database was accessed on 28/04/2022 [4]. Products are authorised on a rolling basis, so the authorised products in the database can change regularly. However, approvals are for up to ten years, so the turnover will be slow. The database was searched twice, once for professional formulations, once for amateur formulations. The web-based table was ported into Excel [5]. The dataset contained formulation name, registration number, authorisation expiry date, marketing company, authorised crops, whether the formulation was authorised in Great Britain, Northern Ireland or both, as well as the active ingredients (but not their concentration).

For adjuvants the same process was used, except as the data was presented over multiple pages, with each page of the web-based table then ported into Excel. Authorisation expiry date, marketing company, authorised crops, and the effective adjuvant component were not included in the aggregated dataset. The effective adjuvant component was only accessible on individual pages, so each page (298 total) was accessed individually, and the effective adjuvant component ported into the Excel file.

### 4.2 Irish datasets

**Fig. 1:**
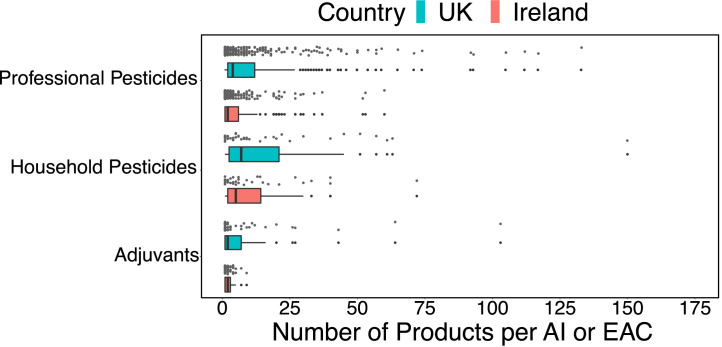
The number of formulations with the same active ingredient (AI) or effective adjuvant component (EAC). Some active ingredients have a large number of formulations (professional: 133 and amateur: 150), while most have less than 25. Professional users include groups like farmers and contract sprayers, amateur users are non-professionals i.e., household and garden use of pesticides.

**Fig. 2:**
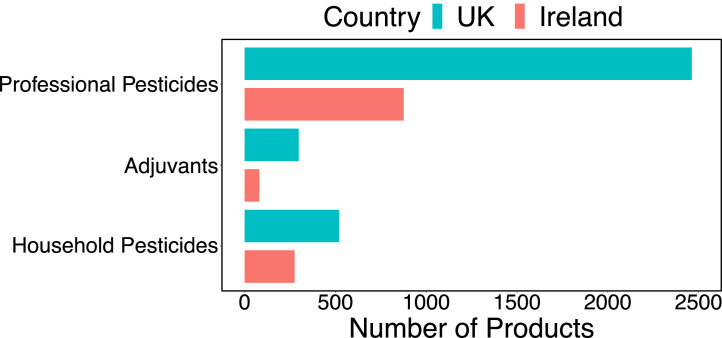
The number of products for professional and amateur pesticide formulations, as well as adjuvant products (only available for professionals).

**Fig. 3:**
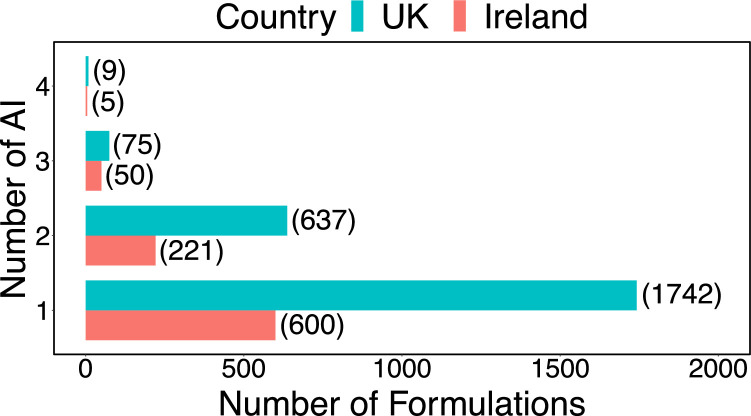
The number of professional pesticide formulations (UK) with one, two, three or four active ingredients (AI). UK data only.

The 2021 datasets containing all authorised pesticide formulations and adjuvants (two separate datasets) was downloaded from the Pesticide Registration and Control Divisions website [6]. The PDF format data was converted to CSV format using Adobe Acrobat Online (Adobe, 2022). The data contained formulation name, registration number, authorisation holder, marketing company, product function, product type, user type and the active ingredients/adjuvant effective components (plus their concentration). User type was not provided for adjuvants as all adjuvants are for professional use, and product function was also not provided for adjuvants. Pesticide formulation data was split into professional and amateur formulations, with a single formulation with both uses included in both datasets (and hence counted in both professional and amateur summary figures). Throughout all figures are for the UK unless otherwise stated (Fig. 1, Fig. 2, Fig. 3).

### 4.3 General

Manual data cleaning was required to standardise the dataset; removing any spelling mistakes and correcting any inconsistent punctuation to ensure consistent reporting. Throughout only obvious spelling errors were corrected, and minor variations between data were assumed to be truthful variation. Company names were written very variably, particularly with additions like country names (e.g. UK) and company types (e.g. Limited). Beyond expanding abbreviations and standardising capitalisation, these were not cleaned, as it is unclear whether the companies operate multiple subsidiaries. As such the number of pesticide producing or marketing companies may be overestimated.

## Limitations

As effective adjuvant components are not regulated with the same level of strictness by the EU (from which the UK derives the vast majority of its current regulation), there is a high likelihood that some effective adjuvant components may be synonyms. Ingredients could also be left off the ingredient list, as legislation governing this is fairly permissive. An example of poor comparability is that alcohol ethoxylates are listed as distinct to alkoylated alcohols in the Irish dataset, despite being nested within them chemically. This was not corrected for, as the literature is too sparse to meaningfully re-categorise them. Broad estimates of diversity are likely be truthful and unchanged by this.

## Ethics Statement

The authors have read and followed the ethical requirements for Data in Brief. This work contains no data on human subjects, animals or data collected from social media platforms.

## CRediT authorship contribution statement

**Edward A. Straw:** Conceptualization, Data curation, Formal analysis, Investigation, Methodology, Validation, Visualization, Writing – original draft, Writing – review & editing. **Dara A. Stanley:** Conceptualization, Methodology, Visualization, Writing – review & editing, Funding acquisition, Project administration.

## Data Availability

A dataset exploring the substantial diversity of pesticide products: insights from the UK and Ireland (Original data) (Zenodo).

## References

[1] Food and Agricultural Organisation (2020). https://www.fao.org/3/cb1329en/CB1329EN.pdf.

[2] Hazen J.L. (2000). Adjuvants – terminology, classification, and chemistry. Weed Technol..

[3] Straw E.A., Stanley D.A. (2023).

[4] Chemical Regulation Division (2022). Available at: https://secure.pesticides.gov.uk/pestreg/prodsearch.asp. Last Accessed in July 2022.

[5] Microsoft Corporation (2018).

[6] Pesticide Registration and Control Division. (2022). Available at: https://www.pcs.agriculture.gov.ie/media/pesticides/content/register/2021book/Plant/20Protection/20Products/202021.pdf. Last Accessed in January 2023.

